# Breastfeeding is associated with waist-to-height ratio in young adults

**DOI:** 10.1186/s12889-015-2611-7

**Published:** 2015-12-23

**Authors:** Adam D. Bohr, Jason D. Boardman, Benjamin W. Domingue, Matthew B. McQueen

**Affiliations:** University of Colorado Boulder, 4185 47th St., Unit C, Boulder, CO 80301 USA; Institute of Behavioral Science, University of Colorado Boulder, 483 UCB, Boulder, CO 80309-0483 USA; Graduate School of Education, Stanford University, 502 Galvez Mall, Stanford, CA 94305 USA; Department of Integrative Physiology, University of Colorado Boulder, 354 UCB, Clare Small 102, Boulder, CO 80309-0354 USA

**Keywords:** Breastfeeding, Obesity, Residual Confounding, Waist-to-Height Ratio

## Abstract

**Background:**

The current study investigated the association between breastfeeding and adult weight distribution using an emerging indicator of weight distribution, the waist-to-height ratio (WHtR).

**Methods:**

The study sample consisted of two subsamples of individuals that were part of the National Longitudinal Study of Adolescent Health. One sample (*n* = 1 179) consisted of individuals from the sibling pair data. A second sample (*n* = 4 648) consisted of individuals that were not part of the paired data. Regression models were constructed to establish if there was a relationship between breastfeeding and two measures of weight distribution: WHtR and body mass index (BMI). Controls for parental socioeconomic status, maternal smoking, race, sex, age, birth weight, maternal BMI, genetic ancestry, and a genetic risk score (GRS) for obesity were included. In addition, a behavioral risk score (BRS) was constructed to control for other residual confounding factors.

**Results:**

A significant, inverse relationship between breastfeeding and adult WHtR persisted in models constructed from the sibling pair sample (*P =* 0.002) and unrelated sample (*P* <0.0001). This association remained significant with the inclusion of ancestry principal components, GRS, and a measure of maternal obesity.

**Conclusions:**

The moderate association between breastfeeding and weight distribution persists into adulthood while controlling for potential confounders. This paper also provides evidence that the WHtR may be a superior outcome measure to BMI in studies investigating breastfeeding and obesity.

**Electronic supplementary material:**

The online version of this article (doi:10.1186/s12889-015-2611-7) contains supplementary material, which is available to authorized users.

## Background

The prevalence of obesity and overweight has increased significantly over the past two decades. Among non-Hispanic white men, the prevalence of obesity increased from 20.3 % in the years 1994–1998 to 35.7 % by 2009–2010 [1]. Recent estimates place the number of overweight at one billion worldwide, of which 300 million are classified as obese [2]. As such, the obesity epidemic has become one of the most pressing public health issues facing global populations.

Increases in obesity prevalence have been linked to an increasingly sedentary lifestyle and consumption of a low quality diet, but these factors alone do not completely explain the nature of the epidemic, nor the fact that some are more prone to becoming obese and retaining weight than others [3, 4]. One of the most important early developmental factors of interest is breastfeeding. A recent study from a sample of overweight adolescents revealed that breastfeeding of an infant is associated with lower incidence of obesity and complications related to metabolic syndrome in the offspring [5]. Other studies have revealed that longer duration of breastfeeding of an infant may be protective against obesity in childhood or reduce the risk of being overweight in childhood [6–9]. Researchers have identified several physiological links between breastfeeding and body size of the offspring. In addition to fatty acids, vitamins, and minerals, breast milk also contains a diverse population of bacteria that colonize the intestinal tract of the infant and may have protective benefits against weight gain throughout life [10]. Breastfeeding duration may also delay the introduction of solid foods for the infant, which has been linked to childhood obesity in some cohorts [11]. However, other studies have found this not to be predictive of obesity [10].

Despite these findings, studies that link breastfeeding to weight status often come under scrutiny. One reason is that the evidence comes solely from observational studies, as randomized trials for breastfeeding would be unethical [12]. The observational studies that do exist have potentially serious problems with selection bias (e.g., those that are most likely to breastfeed may also be the most likely to lead a lifestyle that is related to a healthy body mass index (BMI)) and residual confounding, as breastfeeding is strongly associated with important characteristics of mothers. [6, 12–14] Nevertheless, recent studies have attempted to reduce residual confounding and selection bias by using sibling pairs that were discordant for breastfeeding [15, 16]. They found that the relationship between breastfeeding and BMI observed in between-families analyses was no longer significant in the within-family analysis.

This study builds upon earlier work by using an emerging indicator of weight distribution, the waist-to-height ratio (WHtR), as the primary outcome variable to test this association [17, 18]. There is evidence that the WHtR may be a more accurate diagnostic tool for obesity-related chronic diseases than the more traditional BMI because it more accurately characterizes adiposity [18]. It is known that visceral fat is more metabolic and inflammatory than adipose tissue in other subcutaneous regions [19, 20]. This concept is also supported by a meta-analysis performed by Ashwell et al. (2012) which showed that the WHtR consistently discriminated better for co-morbidities associated with obesity than BMI and waist circumference [17]. Further, in the context of early developmental factors, using height in combination with waist circumference may result in a more sensitive measure to detect early developmental factors associated with obesity. This is because poor fetal nutrition is associated with both short stature and overweight status [18]. We also perform analyses using BMI to allow for comparisons between previously conducted studies of breastfeeding and measures of body mass. Finally, on a subset of the overall sample, we utilize genome-wide association data to incorporate a genetic risk score (GRS) for BMI as well as measures of genetic ancestry.

## Methods

### Participants

The study utilized a sample of individuals from the National Longitudinal Study of Adolescent Health (Add Health) [21]. Add Health is a longitudinal study that investigates how social and environmental factors may influence health. It has followed a cohort of individuals through four waves of interviewing and testing since its inception during the 1994–1995 school year. The full Add Health study consisted of 20 792 participants. However, a substantial amount of this sample was not part of the Wave 1 parent interviews or measured for waist circumference and height at Wave 4. In addition, genome-wide association data is available on the sibling pair (SP) sample only. Therefore, we analyzed a subsample of 1 179 respondents with genome-wide data from the SP sample as well as 4 648 respondents from the unrelated (UN) sample. Also, to ensure that the relatedness of the siblings was not impacting the associations observed in the SP sample, we randomly selected one participant from each sibling pair resulting in a third sample of 856 respondents and performed analyses on this sample. Variables from all four waves of interviews and measurements from the Add Health Study were used. In an effort to retain the largest possible sample size, missingness was allowed for two covariates in the analysis: birth weight and parental smoking.

### Data

The specific Add Health variable code names used in this study are listed in the supplementary information section to this paper (Additional file 2: Table S1). The primary variables of interest in this study were breastfeeding, WHtR, and BMI. Breastfeeding data were extracted from the in-home parent interviews conducted during Wave 1 (baseline). The options provided for the breastfeeding question were “Never Breastfed”, “0–3 Months”, “3–6 Months”, “6–9 Months”, “9–12 Months”, “12–24 Months”, and “Greater than 24 Months.” We removed any cases of missingness for breastfeeding from our analyses. In an effort to reduce any potential recall bias that may exist in the reporting of duration of breastfeeding, we opted to analyze breastfeeding only as a dichotomous variable. Individuals that were never breastfed were coded as “0” while those that were breastfed were coded as “1”.

Height (cm) and waist circumference (cm) were extracted from the Add Health Wave 4 anthropometric measurements. Waist circumference was measured to the nearest 0.5 cm at the superior border of the iliac crest. [21] The WHtR metric was calculated by dividing waist circumference by height. BMI values were also taken from Wave 4 data. As with breastfeeding, we removed any cases from our analyses that were missing for WHtR and BMI.

To control for behavioral differences between individuals, we utilized risk scores constructed from a variety of indicators. The risk scores were calculated using the R package, pcaMethods, and allowed for missing data [22]. For this study, the behavioral risk scores (BRS) are the first three principal components derived from a cluster of items regarding diet, sleep, and exercise. The BRS components were validated using both Wave 4 WHtR and BMI. The specific Add Health variables used and details on how the risk scores were constructed can be found in the supplementary information (Additional file 1 and Additional file 2: Table S2).

A genetic risk score (GRS) was calculated for each individual in the SP data set. Genome-wide association studies have identified 31 single nucleotide polymorphisms (SNPs) in individuals of European descent and 8 SNPs in individuals of African American descent that are associated with BMI [23, 24]. These SNPs were used to compute risks scores, and analyses were conducted testing the association of these risk scores with BMI, obesity, WHtR, and change in BMI over time. A more in depth description of these risk scores and their construction using the SP sample can be found in Domingue et al. [25]. To develop our measure of genetic ancestry, we used KING to identify clusters of individuals based upon genetic similarity [26]. KING uses multidimensional scaling (MDS) with Euclidean distance to generate principal coordinates (PCs) that can be used to identify population substructure. A full description of the derivation of the ancestry PCs can be found in McQueen et al. [27].

In addition to the calculated risk scores, we used the following controls: birth weight (lb.); parental SES; parental smoking; maternal obesity; gender; age; and race. Birth weights for the subjects were obtained from the Wave 1 in-home parent interviews. Because low birth weight is hypothesized to be associated with higher WHtR in adulthood, birth weight was assessed as a categorical variable that compared low birth weight subjects to normal birth weight subjects. Low birth weight status was assigned to subjects that weighed less than 2 500 g (5.5 lb.). As stated above, this variable allowed for missingness and was analyzed as a factor, with low birth weight and missing birth weight compared to the referent category, normal birth weight.

Self-reported parental smoking status was also extracted from the Wave 1 in-home parent interviews and analyzed as a factor, allowing for missingness, with “yes” for smoking and missingness for smoking compared to the referent group of nonsmoking. For a measure of maternal obesity, we used the question “Is the biological mother obese?” from the Wave 1 in-home parent interview. This variable was dichotomous, and a response of “no” was coded as “0” while “yes” was coded as “1”. Self-reported race was analyzed as a factor, comparing Hispanic, non-Hispanic white, and non-Hispanic Asian/Native American to the referent group non-Hispanic African American. Parental SES was assessed with self-reported household income at Wave 1 of the study and was analyzed as a dichotomous variable. We coded those that reported income less than the median income from the overall sample ($38 000) as low SES (“0”) and those that reported income of greater than the median as high SES (“1”).

Descriptive statistics in total and stratified by breastfeeding status for sex, race, parental smoking, low birth weight occurrence, parental SES, maternal obesity, BMI, and WHtR for the two samples can be found in Tables 1 and 2. The average age of the participants at Wave 4 was 28.49 years with a range of 24.32–33.59 years. Breastfeeding initiation was 49.2 % in our UN sample and 39.4 % in our SP sample. In reviewing the descriptive statistics for this study, it is worth commenting on certain elements of the sample. Rates of breastfeeding initiation were 26.5 % in 1970 and rose to 61.9 % in 1982 [28]. As the participants in the Add Health study were born between 1976 and 1984, it is reasonable that the initiation rates in our samples would be between these two rates. In addition, there are some racial disparities in the initiation of breastfeeding. African Americans comprise 18.4 % of the individuals in the UN sample but only 11.68 % of the individuals that were breastfed. This disparity was observed in the SP sample as well. This is consistent with previous literature that has demonstrated a lower rate of breastfeeding initiation in African Americans, while there are not large discrepancies between non-Hispanic white or Hispanics [29]. In addition, it may explain some of the difference in breastfeeding rates between our two samples, as the SP sample was 34.18 % African American and the UN sample was 18.42 % African American. Finally, there were differences in the rates of low birth weight between the two samples (UN Sample: 4.65 %; SP Sample: 13.57 %) that are likely attributed to the oversampling of African Americans.

**Table 1:** Descriptive statistics for participants from the add health study, 1994-2008. Unrelated sample

			Breastfeeding status (Yes/No)			
	Full sample		No		Yes	
	4648		2362		2286	
	n	%	n	%	n	%
Males	2116	45.52 %	1056	44.71 %	1060	46.37 %
Females	2532	54.48 %	1306	55.29 %	1226	53.63 %
Race						
Non-Hispanic White	2865	61.64 %	1368	57.92 %	1497	65.49 %
Non-Hispanic African American	856	18.42 %	589	24.94 %	267	11.68 %
Non-Hispanic Asian/Native American	259	5.57 %	89	3.77 %	170	7.44 %
Hispanic	666	14.33 %	315	13.34 %	351	15.35 %
Parent smoker						
No	714	15.36 %	370	15.66 %	344	15.05 %
Yes	1328	28.57 %	838	35.48 %	490	21.43 %
Missing	2606	56.07 %	1154	48.86 %	1452	63.52 %
Low birth weight						
No	4351	93.61 %	2155	91.24 %	2196	96.06 %
Yes	216	4.65 %	146	6.18 %	70	3.06 %
Missing	81	1.74 %	61	2.58 %	20	0.87 %
Parent socioeconomic status						
Below median income	2101	45.20 %	1250	52.92 %	851	37.23 %
Above median income	2547	54.80 %	1112	47.08 %	1435	62.77 %
Maternal obesity						
No	3790	81.54 %	1901	80.48 %	1889	82.63 %
Yes	858	18.46 %	461	19.52 %	397	17.37 %
	Mean	SE	Mean	SE	Mean	SE
Body mass index	29.007	0.112	29.803	0.165	28.185	0.148
Waist-to-height ratio	0.575	0.002	0.589	0.002	0.562	0.002

**Table 2:** Descriptive statistics for participants from the Add Health Study, 1994–2008. Sibling pair sample

			Breastfeeding status (Yes/No)			
	Full sample		No		Yes	
	1179		715		464	
	n	%	n	%	n	%
Males	581	49.28 %	350	48.95 %	231	49.78 %
Females	598	50.72 %	365	51.05 %	233	50.22 %
Race						
Non-Hispanic White	625	53.01 %	321	44.90 %	304	65.52 %
Non-Hispanic African American	403	34.18 %	333	46.57 %	70	15.09 %
Non-Hispanic Asian/Native American	35	2.97 %	8	1.12 %	27	5.82 %
Hispanic	116	9.84 %	53	7.41 %	63	13.58 %
Parent smoker						
No	194	16.45 %	116	16.22 %	78	16.81 %
Yes	369	31.30 %	270	37.76 %	99	21.34 %
Missing	616	52.25 %	329	46.01 %	287	61.85 %
Low birth weight						
No	984	83.46 %	558	78.04 %	426	91.81 %
Yes	160	13.57 %	126	17.62 %	34	7.33 %
Missing	35	2.97 %	31	4.34 %	4	0.86 %
Parent socioeconomic status						
Below median income	661	56.06 %	453	63.36 %	208	44.83 %
Above median income	518	43.94 %	262	36.64 %	256	55.17 %
Maternal obesity						
No	909	77.10 %	560	78.32 %	349	75.22 %
Yes	270	22.90 %	155	21.68 %	115	24.78 %
	Mean	SE	Mean	SE	Mean	SE
Body mass index	29.321	0.223	29.880	0.300	28.459	0.325
Waist-to-height ratio	0.580	0.003	0.588	0.004	0.566	0.004

### Statistical analysis

#### Unrelated sample (UN)

OLS regression models were used to investigate the relationship between breastfeeding and measures of weight distribution. For both WHtR and BMI, breastfeeding was the primary predictor variable of interest. As noted above, breastfeeding was analyzed as a dichotomous variable (ever breastfed coded as “1”, never breastfed coded as “0”) in these models. Models constructed from the UN data included all covariates with the exception of the genetic risk scores and ancestry principal components. We also tested for a maternal obesity and breastfeeding interaction to assess whether or not the association between breastfeeding and weight distribution was dependent on the weight status of the mother. This interaction term was not significant in any of our analyses and therefore excluded from the final models. Additionally, we tested for an interaction between breastfeeding and the genetic risk score that was also not significant and excluded from the final models. Finally, we used the Add Health Wave 1 sample weights for all models in the UN sample. The statistical power for these models was as follows: WHtR Outcome, (1 - β)  <0.001; BMI Outcome (1 - β)  <0.001.

In addition to the linear models, we also constructed logistic regression models to further investigate the decrease in risk of obesity associated with breastfeeding in both samples. For this analysis, we created a dichotomous variable to indicate obesity status (BMI ≥ 30). All analyses and calculations were done using R version 2.15.3 via the RStudio platform, version 0.97.320 [30].

#### Sibling Pair Sample (SP)

OLS regression models were also constructed from the SP sample. These models included all of the covariates that were tested in the UN sample and tested the outcomes WHtR, BMI, and BMI ≥ 30. In addition, these models included the PCs for genetic ancestry and the GRS as controls. The statistical power for these models was as follows: WHtR Outcome, (1 - β)  = 0.018; BMI Outcome (1 - β)  = 0.177.

#### Subset of Sibling Pair Sample (SSP)

Finally, OLS regression models were also constructed from a subset of the SP sample. One participant was randomly selected from each of the sibling pairs, and the OLS models described for the SP sample were also constructed from this subset. The statistical power for these models was as follows: WHtR Outcome, (1 - β)  = 0.093; BMI Outcome (1 - β)  = 0.422.

## Results

### Primary outcomes and exposures

The results of the multiple linear regression models from the UN and SP samples are presented in Tables 3 and 4. In our UN sample, breastfeeding was associated with a decrease in both WHtR and BMI. All other factors being equal, breastfeeding was associated with a decrease in predicted adult WHtR of 0.012 and in predicted adult BMI of 0.548. We observed a consistent effect in the SP sample, as breastfeeding was associated with a decrease in both WHtR and BMI. Once again, all other factors being equal, breastfeeding was associated with a decrease in predicted adult WHtR of 0.020 and predicted adult BMI of 1.049. As anticipated, the GRS is a highly significant predictor of body weight measures in all models constructed from the SP sample. The third principal component for ancestry was a significant predictor of both WHtR and BMI in the SP sample while the fifth principal component was predictive of BMI only. All three principal components of the BRS were significant predictors of body weight measures in our UN sample, but only the first principal component remained significant with the inclusion of the GRS in the SP sample.

**Table 3:** Regression results for models constructed from unrelated sample, *n* = 4648, Add Health Study 1994–2008

	Waist-to-height ratio outcome*			Body mass index outcome**		
	b	SE	*P* value	b	SE	*P* value
Intercept	0.541	0.027	<0.0001	29.085	2.082	<0.0001
Sex	0.028	0.003	<0.0001	−0.061	0.220	0.783
Age	0.001	0.001	0.516	0.075	0.069	0.278
Breastfeeding	−0.012	0.003	<0.0001	−0.516	0.221	0.020
Birthweight >2500 g	Reference	Reference	Reference	Reference	Reference	Reference
Birthweight <2500 g	0.001	0.006	0.926	−0.965	0.485	0.047
Birthweight missing	0.009	0.011	0.427	−0.298	0.829	0.719
Parent nonsmoker	Reference	Reference	Reference	Reference	Reference	Reference
Parent smoker	0.010	0.004	0.024	0.808	0.327	0.014
Parent smoking missing	−0.011	0.004	0.005	−0.769	0.303	0.011
Non-Hispanic African American	Reference	Reference	Reference	Reference	Reference	Reference
Non-Hispanic White	−0.013	0.004	0.003	−1.911	0.330	<0.0001
Non-Hispanic Asian/Native American	−0.014	0.009	0.115	−2.185	0.671	0.001
Hispanic	0.010	0.006	0.081	−0.292	0.430	0.498
Behavioral risk score 4_1	−0.015	0.002	<0.0001	−0.773	0.125	<0.0001
Behavioral risk score 4_2	−0.007	0.003	0.007	−0.399	0.192	0.038
Behavioral risk score 4_3	−0.008	0.003	0.003	−0.377	0.197	0.055
Parent socioeconomic status	−0.002	0.001	<0.0001	−0.141	0.043	0.001
Maternal obesity	0.055	0.004	<0.0001	4.525	0.271	<0.0001

b (Coefficient) SE (Standard Error)

* R-Squared = 0.13

** R-Squared = 0.10

**Table 4:** Regression results for models constructed from sibling pair sample, *n* = 1179, Add Health Study 1994–2008

	Waist-to-height ratio outcome*			Body mass index outcome**		
	b	SE	*P* value	b	SE	*P* value
Intercept	0.392	0.063	<0.0001	20.161	4.640	<0.0001
Sex	0.037	0.006	<0.0001	0.301	0.477	0.528
Age	0.000	0.002	0.838	−0.063	0.132	0.634
Breastfeeding	−0.020	0.006	0.002	−1.049	0.473	0.027
Birthweight >2500 g	Reference	Reference	Reference	Reference	Reference	Reference
Birthweight <2500 g	0.001	0.008	0.927	−0.186	0.629	0.768
Birthweight Missing	0.006	0.017	0.726	0.384	1.261	0.761
Parent nonsmoker	Reference	Reference	Reference	Reference	Reference	Reference
Parent smoker	0.008	0.009	0.334	0.322	0.648	0.619
Parent smoking missing	0.007	0.008	0.375	0.686	0.598	0.251
Non-Hispanic African American	Reference	Reference	Reference	Reference	Reference	Reference
Non-Hispanic White	−0.014	0.027	0.604	−1.634	2.004	0.415
Non-Hispanic Asian/Native American	0.025	0.047	0.589	3.625	3.452	0.294
Hispanic	−0.023	0.026	0.382	−1.695	1.918	0.377
Behavioral risk score 4_1	0.016	0.004	<0.0001	0.757	0.265	0.004
Behavioral risk score 4_2	−0.001	0.004	0.787	−0.206	0.282	0.466
Behavioral risk score 4_3	−0.002	0.004	0.564	0.123	0.303	0.685
Parent socioeconomic status	−0.010	0.006	0.100	−0.839	0.462	0.070
Maternal obesity	0.047	0.007	<0.0001	3.932	0.508	<0.0001
Genetic risk score	0.005	0.001	<0.0001	0.402	0.065	<0.0001
Ancestry principal component 1	0.273	0.544	0.617	10.394	40.325	0.797
Ancestry principal component 2	−0.392	0.348	0.261	−4.349	25.792	0.866
Ancestry principal component 3	0.769	0.215	<0.001	57.713	15.944	<0.001
Ancestry principal component 4	−0.034	0.150	0.822	0.989	11.076	0.929
Ancestry principal component 5	0.366	0.187	0.0505	32.468	13.836	0.0191

b (Coefficient) SE (Standard Error)

* R-Squared = 0.18

** R-Squared = 0.14

The results from the logistic regression model are presented in Table 5 and include calculated odds ratios, 95 % confidence intervals, and *P* values. Consistent with the results of the linear models, breastfeeding was associated with a decreased risk of obesity in the UN sample (OR = 0.854, 95 % CI = (0.75, 0.97)). With all other factors being equal, having been breastfed was associated with a 0.854 odds of obesity compared to those that were not breastfed. Conversely, those that were not breastfed were 1.171 times more likely to be obese than those that were breastfed with all else being equal. Breastfeeding was associated with the same odds ratio in the SP sample, though it was not statistically significant (OR = 0.854, 95 % CI = (0.64, 1.13)).

**Table 5:** Results of logistic regression predicting BMI ≥ 30: Unrelated and sibling pair sample, Add Health Study, 1994–2008

	Unrelated sample			Sibling pair sample*		
	OR	95 % CI	*P* value	OR	95 % CI	*P* value
Sex	0.991	(0.87, 1.13)	0.897	1.114	(0.84, 1.48)	0.451
Age	1.032	(0.99, 1.08)	0.140	1.021	(0.94, 1.10)	0.613
Breastfeeding	0.854	(0.75, 0.97)	0.018	0.854	(0.64, 1.13)	0.277
Birthweight >2500 g	Reference	Reference	Reference	Reference	Reference	Reference
Birthweight <2500 g	1.073	(0.80, 1.44)	0.635	1.162	(0.81, 1.67)	0.419
Birthweight missing	0.614	(0.37, 1.01)	0.055	0.908	(0.43, 1.93)	0.801
Parent nonsmoker	Reference	Reference	Reference	Reference	Reference	Reference
Parent smoker	1.259	(1.04, 1.53)	0.021	0.909	(0.62, 1.33)	0.623
Parent smoking missing	0.863	(0.72, 1.03)	0.106	0.874	(0.61, 1.25)	0.456
Non-Hispanic African American	Reference	Reference	Reference	Reference	Reference	Reference
Non-Hispanic White	0.665	(0.56, 0.79)	<0.0001	0.439	(0.14, 1.41)	0.168
Non-Hispanic Asian/Native American	0.528	(0.38, 0.73)	<0.0001	0.682	(0.08, 5.83)	0.727
Hispanic	1.055	(0.85, 1.31)	0.621	0.738	(0.24, 2.27)	0.596
Behavioral risk score 4_1	0.794	(0.73, 0.86)	<0.0001	1.244	(1.06, 1.47)	0.009
Behavioral risk score 4_2	0.834	(0.75, 0.93)	0.002	0.976	(0.83, 1.15)	0.779
Behavioral risk score 4_3	0.867	(0.77, 0.98)	0.018	0.904	(0.75, 1.09)	0.283
Parent socioeconomic status	0.960	(0.94, 0.98)	0.002	0.652	(0.49, 0.86)	0.003
Maternal obesity	2.539	(2.17, 2.97)	<0.0001	2.794	(2.08, 3.75)	<0.0001
Genetic risk score	–	–	–	1.092	(1.05, 1.14)	<0.0001

OR (Odds Ratio), 95 % CI (95 % Confidence Interval)

*The analyses for the paired sample also controlled for genetic ancestry which is not displayed in the above table

The results of the multiple linear regression models and logistic regression model constructed from the subset of the SP sample are presented in Table 6. Consistent with the results from both the UN sample and full SP sample, breastfeeding was associated with a decrease in both WHtR and BMI. All other factors being equal, breastfeeding was associated with a decrease in predicted adult WHtR of 0.025 and in predicted adult BMI of 1.604. In addition, the results of the logistic regression predicting BMI ≥ 30 showed a significant association with breastfeeding (OR = 0.695, 95 % CI = (0.50, 0.97)). With all other factors being equal, those that were not breastfed were 1.43 times more likely to be obese than those that were breastfed.

**Table 6:** Results of regression models predicting waist-to-height ratio, body mass index, and obesity from randomly selected subset of the sibling pair sample, *n* = 856, Add Health Study 1994–2008

	Waist-to-height ratio outcome*			Body mass index outcome**			Body mass index ≥ 30 outcome***		
	b	SE	*P* value	b	SE	*P* value	OR	95 % CI	*P* value
Intercept	0.415	0.067	<0.0001	19.782	5.060	<0.0001	0	0	0
Sex	0.042	0.007	<0.0001	0.564	0.524	0.282	1.127	(0.83, 1.53)	0.439
Age	−0.001	0.002	0.665	−0.045	0.147	0.762	0.993	(0.91, 1.08)	0.863
Breastfeeding	−0.025	0.008	0.001	−1.604	0.576	0.005	0.695	(0.50, 0.97)	0.033
Birthweight >2500 g	Reference	Reference	Reference	Reference	Reference	Reference	Reference	Reference	Reference
Birthweight <2500 g	0.006	0.010	0.524	0.174	0.749	0.816	1.213	(0.80, 1.85)	0.369
Birthweight Missing	0.011	0.019	0.563	0.988	1.460	0.499	1.129	(0.49, 2.58)	0.774
Parent nonsmoker	Reference	Reference	Reference	Reference	Reference	Reference	Reference	Reference	Reference
Parent smoker	0.005	0.010	0.619	0.318	0.784	0.685	0.955	(0.61, 1.50)	0.840
Parent smoking missing	0.005	0.010	0.625	0.724	0.726	0.319	0.998	(0.66, 1.52)	0.994
Non-Hispanic African American	Reference	Reference	Reference	Reference	Reference	Reference	Reference	Reference	Reference
Non-Hispanic White	−0.022	0.030	0.455	−2.317	2.228	0.299	0.382	(0.11, 1.37)	0.140
Non-Hispanic Asian/Native American	0.022	0.053	0.682	2.978	3.954	0.452	0.378	(0.3, 4.90)	0.456
Hispanic	−0.034	0.028	0.227	−2.587	2.138	0.227	0.594	(0.17, 2.03)	0.406
Behavioral risk score 4_1	−0.013	0.004	0.001	−0.534	0.305	0.080	0.827	(0.69, 0.99)	0.044
Behavioral risk score 4_2	0.000	0.004	0.990	−0.044	0.328	0.894	1.038	(0.85, 1.27)	0.713
Behavioral risk score 4_3	0.003	0.006	0.616	0.296	0.419	0.480	1.174	(0.92, 1.50)	0.200
Parent socioeconomic status	−0.017	0.008	0.025	−0.975	0.567	0.086	0.653	(0.47, 0.91)	0.011
Maternal obesity	0.039	0.008	<0.0001	3.339	0.619	<0.0001	2.433	(1.72, 3.45)	<0.0001
Genetic risk score	0.005	0.001	<0.0001	0.412	0.080	<0.0001	1.079	(1.03, 1.13)	0.001
Ancestry principal component 1	0.575	0.599	0.338	36.065	45.094	0.424	0	0	0
Ancestry principal component 2	−0.416	0.398	0.297	−8.198	29.971	0.785	0	0	0
Ancestry principal component 3	0.982	0.237	<0.001	67.069	17.867	<0.001	0	0	0
Ancestry principal component 4	0.008	0.164	0.961	4.257	12.316	0.730	0	0	0
Ancestry principal component 5	0.346	0.205	0.092	29.949	15.419	0.052	0	0	0

b (Coefficient) SE (Standard Error) OR (Odds Ratio) 95 % CI (95 % Confidence Interval)

* R-Squared = 0.18

** R-Squared = 0.13

*** This analysis also controlled for genetic ancestry which is not displayed in the above table

### Other covariates

Low birth weight was associated with decreased BMI in our UN sample but was not a significant predictor in any other models. Parental smoking was a significant predictor of both BMI, WHtR, and BMI ≥ 30 in the UN sample, but was not significant in the SP sample. Several of our racial categories were significant predictors of BMI in the UN sample. Reporting non-Hispanic white or non-Hispanic Asian/Native American was associated with a decrease in predicted BMI when compared with the referent group, non-Hispanic African American. However, this effect was only seen for those reporting white for the WHtR outcome. Of particular note, race was not a significant predictor in any of models from the SP sample or the subset from the SP sample. Our measure of parental SES was inversely related to WHtR and BMI in the UN sample and was trending in the SP sample. Parental SES was also a significant predictor of WHtR and was trending for BMI in the subset of the SP sample. Our variable for maternal obesity was a strong positive predictor of BMI, WHtR, and BMI ≥ 30 in all samples.

## Discussion

Previous research has linked a variety of early childhood factors to the health status of adults [31]. A great deal of recent interest has focused on breastfeeding as an important determinant of health status into adulthood, and our study contributes to this body of work in several important ways. Few studies investigating the role breastfeeding use the WHtR as an outcome as opposed to BMI, and to our knowledge none have done so with adults. This is important because, while there are debates about the value of the BMI in epidemiologic research, the WHtR has been shown to be a superior indicator of physical composition as it describes the distribution of weight. [17–20] As such the inclusion of this measure enhances our understanding of the role of breastfeeding for the health of populations.

Second, the Add Health study has several features that enhance the validity of our findings. For example, BMI and WHtR were both measured in the field by trained technicians rather than self-reported. The design of the Add Health study allows for opportunity to make true population inferences not only about prevalence and incidence but more importantly about key associational parameters such as breastfeeding and adult health. The richness of these data allow us to include detailed and comprehensive measures of behavioral and environmental risks, such as SES, diet, and exercise, that may confound the association. In addition, our regression models had controls in place for race, parental SES, parental smoking, maternal obesity, and low birth weight. Finally, controlling for maternal obesity provides additional support that the association between breastfeeding and weight distribution is not dependent on family factors.

Finally, we were able to establish a consistent effect of breastfeeding on both WHtR and BMI in both Add Health subsamples. This finding was robust in the SP data set that included genetic risk factors for BMI/obesity as well as measures of genetic ancestry. We further tested this finding on a subset of the SP sample, demonstrating that this association was not due to the relatedness of the subjects. To our knowledge, this is the first study to assess the role of breastfeeding on weight distribution using measured genetic factors that could influence the risk of obesity. The robustness of the association between breastfeeding and measures of weight status controlling for genetics is novel and provides additional support for the association between breastfeeding and weight distribution that has been reported elsewhere.

There are many characteristics of breastfeeding that may help to explain how feeding practice early in life can influence weight distribution and possibly have protective benefits against obesity in adulthood. To begin, breastfeeding appears to result in lower overall energy intake than formula feeding in the early postnatal period and is associated with an infant’s ability to self-regulate milk intake [32, 33]. These findings take on greater significance when one considers the effect that early weight gain can have on weight status in later childhood and even adulthood. There is evidence that overweight status in childhood has an impact on the risk of being overweight in adulthood [33]. In addition, rapid weight gain in infancy is related to being overweight later in childhood [34].

A recent study that is most comparable to the current study was conducted by Rousseaux et al. [35]. The study used multivariate analysis to test for relationships with measures of body composition including WHtR. It found no significant associations between breastfeeding and measures of body composition. However, a nonsignificant trend towards a protective benefit was identified on the highest percentiles of abdominal adiposity. The two studies differed in the age of the participants in addition to a large difference in mean WHtR, which could account for the disparity in the findings. Given that HELENA is a cohort study, future research is warranted to test for the association in adulthood.

A previous study that is in conflict with the current study was a randomized control trial conducted in Belarus. This trial was a breastfeeding promotion intervention, and found that the intervention increased the duration of breastfeeding but did not influence weight or adiposity at roughly 6.5 years old [12]. However, the study was conducted in Belarus, a country that does not have a comparable prevalence of obesity to the United States. Therefore, the results may not be generalizable to the cohort used in the current study. In addition, this study influenced only duration of breastfeeding and not initiation. The current study compared individuals that were breastfed to those that were not breastfed. It may be possible that while long duration of breastfeeding doesn’t add any additional risk reduction benefit, breastfeeding in early infancy may convey a significant benefit vs. individuals that were never breastfed at all.

Another study that contrasted with ours and used the Add Health sample was conducted by Evenhouse and Reilly.  It found no differences in BMI between sibling pairs discordant for breastfeeding [15]. Utilizing a within-family discordant sibling model is an efficient approach to control for family-level effects including genetic ancestry, race, and SES. However, there are other differences between the two studies that could account for the contrasting findings. First, within-family analyses using discordant sibling pairs can be limited by within-family variation in breastfeeding. As noted in Evanhouse and Reilly, there were 523 sibling pairs discordant for breastfeeding *duration* and only 288 pairs discordant for breastfeeding (one sibling breastfed, the other did not). Given the relatively high correlation of BMI within sibling pairs (heritability estimates range from 60 to 80 %), it is possible that a discordant sibling pair study was underpowered to detect an association due to breastfeeding [36, 37]. Additionally, the present study used the participants BMI and WHtR at Wave 4 as opposed to Wave 1, which would have reduced our sample size for pairs discordant for breastfeeding initiation to 62 and substantially reduced our statistical power to detect any differences between siblings discordant for breastfeeding. Finally, we observed that WHtR may be a more sensitive measure of body weight compared to BMI as it relates to the association with breastfeeding.

### Limitations

One of the primary criticisms of breastfeeding studies in general is related to residual confounding and recall bias. In this study, we were not able to control for maternal diet, which is often implicated in early infant weight gain and childhood obesity. In addition, the information on breastfeeding duration was via self-report from the mothers. On average, mothers would have been asked about breastfeeding duration approximately 12–15 years after the breastfeeding took place. It is unclear as to whether recall biases may exist in this regard. However, there aren’t any reasons to expect systematic differences in recall for those individuals who have overweight children vs. those that do not. In addition, we analyzed breastfeeding strictly as an ever/never variable. While this has the detrimental effect of not discriminating between infants that were breastfed for a day and those that were breastfed for two years, it may have minimized some of the recall bias. Also, our control for maternal obesity was a self-reported measure at the time of the questionnaire. While this isn’t an ideal measure of maternal obesity at the time of the child’s birth, the rate of obesity in the UN sample (18.46 %) is consistent with NHANES measures from 1976 to 1980 (17.1 %) [38], and we feel that the mother’s weight status at the time of the parental survey may serve as an appropriate proxy for her status during and after pregnancy. Finally, we did not have information on whether or not the reported breastfeeding duration was exclusive or partial, and it is possible that the relationship between weight distribution and breastfeeding status could be affected by whether or not the breastfeeding was supplemented with solid food.

The genetic risk score consisted of 31 SNPs that had been validated using a sample of both black and white young adults from the Add Health Study [25]. As such, these SNPs were used to construct the genetic risk score in the current study. Genome-wide association studies have identified 90+ SNPs that are associated with body mass/obesity. However, for the purposes of this study, we were not focused on developing an optimal set of SNPs to predict body mass/obesity. Rather, our analysis was focused on an established set of SNPs that sufficiently accounts for the genetic risk of obesity [39–42]. While additional SNPs (90+ or including genome-wide SNPs) would result in a slightly better genetic risk score prediction, we are confident that adding the very small effects of these additional SNPs would not impact our primary analytic aim: relating breastfeeding to WHtR. Additionally, the measures of genetic ancestry were included to adjust our analyses for potential genetic (as opposed to racial/ethnic) differences on the genome-wide scale.

Finally, the analysis was limited due to the availability of the genetic measures in the sibling pair sample. We tested for whether or not the relationship we observed between breastfeeding and measures of weight status in the sibling sample was an artifact of the relatedness of the participants. We randomly sampled one sibling from each pair and conducted the analyses on these participants. In all follow-up analyses, we observed the consistent, significant relationship between breastfeeding and measures of weight status. While other methods may be more efficient in the context of biologically related individuals (i.e., mixed effects regression) we were not interested in studying the family-level effects in these analyses. Furthermore, there are a limited number of sibling pairs discordant for breastfeeding, which diminishes our ability to assess any family-level impact on the association of interest.

## Conclusion

Overall, we contend that our collective attention to race, SES, GRS, maternal obesity, and BRS would broadly serve as correlates of various lifestyle and body weight indicators. Residual confounding undoubtedly remains in these analyses. However, there would have to be substantial unmeasured confounding influence to be able to explain enough of the relationship between breastfeeding and weight distribution to completely ameliorate the associations observed in this study.

In conclusion, the results of the current study demonstrated that breastfeeding is inversely related to the WHtR in adulthood. While many studies have reported a relationship between breastfeeding duration and weight in childhood, few others have reported this association in a young adult population. Also the current study provided evidence that WHtR may be a more sensitive outcome variable than BMI for studies that investigate the association between breastfeeding and weight distribution or status. Finally, the current study demonstrated both the utility of using genetic factors underlying obesity as well as evaluating the robustness of the breastfeeding association controlling for these factors. We encourage future studies to investigate the mechanisms that may be responsible for the role of breastfeeding on weight distribution.

### Data availability statement

All data used in this study is publicly available at the Add Health website: www.cpc.unc.edu/projects/addhealth.

### Supplementary information

Supplementary information is available at BMC Public Health’s website.

## Additional files

Additional file 1:
**Supplementary information.** (DOCX 99 kb) Additional file 2:
**Table S1.** Regression Model Covariates. **Table S2.** Behavioral Risk Score Variables. (XLSX 50 kb)

## Abbreviations

BMI: body mass index
BRS: behavioral risk score
GRS: genetic risk score
PC: principal component
SES: socioeconomic status
SP: sibling pair sample
UN: unrelated sample
WHtR: waist-to-height ratio
