# Supplementary material for: Breastfeeding is associated with waist-to-height ratio in young adults
Source: BMC Public Health. 2015 Dec 23;15:1281. doi: 10.1186/s12889-015-2611-7 (PMC4688938; doi:10.1186/s12889-015-2611-7)
Supplement: Additional file 1: — Supplementary information. (DOCX 99 kb) [file 12889_2015_2611_MOESM1_ESM.docx]

**Supplementary Information**

As discussed in the data section of the manuscript, risk scores were constructed to control for behavioral differences between the participants that could account for variance in the outcome variables of interest. These scores were constructed from a variety of variables describing participants’ daily activity, diet, and sleep behaviors. The supplementary section to this paper will provide additional information on the construction, validation, and variables used for these risk scores. The phenotypic measures that were used for this paper were Wave 4 BMI and Wave 4 WHtR. For the phenotypic principal component, missing data was allowed. However, an individual had to have values for at least half the relevant items to receive principal component estimates. The control variables we included in the validation analyses are controls for race, sex, SES, parental education, and whether an individual lived with their mother. The specific Add Health variables we used for the construction of the risk scores are listed in Supplementary Tables 2.

Once the risk scores were derived, regression analysis was used to test the association between our phenotypic variables of interest and the constructed risk scores. The first behavioral risk score (BRS) principal component was able to account for approximately 1.1% and 3.5% of the variance seen in Wave 4 BMI and WHtR, respectively. The addition of the next two BRS principal components only increased the explained variance to approximately 1.7% and 3.6% for Wave 4 BMI and WHtR.

**References for Supplementary Information**

1. Boardman JD, Roettger ME, Domingue BW, McQueen MB, Haberstick BC, Harris KM. Gene-environment interactions related to body mass: School policies and social context as environmental moderators. *Journal of Theoretical Politics*. 2012;24(3): 370–388.
2. Gordon-Larsen, P. *ONEdata-Obesity & Neighborhood Environment Database.*  <http://www.cpc.unc.edu/projects/onedata>.
